# Supplementary material for: Altered microRNA Transcriptome in Cultured Human Liver Cells upon Infection with Ebola Virus
Source: Int J Mol Sci. 2021 Apr 6;22(7):3792. doi: 10.3390/ijms22073792 (PMC8038836; doi:10.3390/ijms22073792)
Supplement: Supplementary file 1 [file ijms-22-03792-s001.zip › Supplementary_File/C_ GO_Analysis_Results/16-30nt_go_Reston-24h-Huh7_vs_Control-24h-Huh7_up.mature_mirna_targets/GeneOntology_FoldEnrichment.pdf]

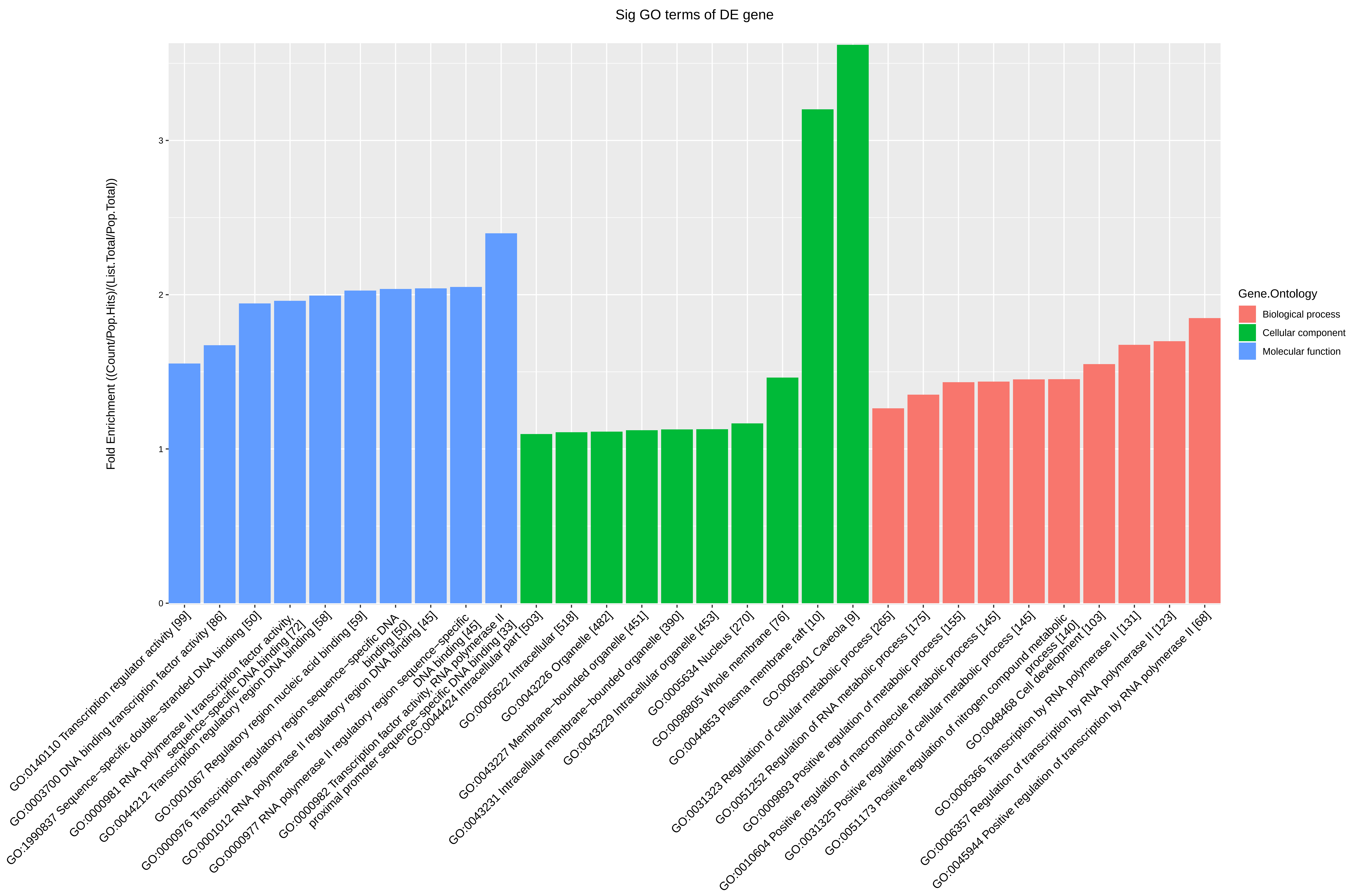

Sig GO terms of DE gene
Fold Enrichment ((Count/Pop.Hits)/(List.Total/Pop.Total))
0
1
2
3
Gene.Ontology
Biological process
Cellular component
Molecular function
GO:0140110 Transcription regulator activity [99]
GO:0003700 DNA binding transcription factor activity [86]
GO:1990837 Sequence−specific double−stranded DNA binding [50]
GO:0000981 RNA polymerase II transcription factor activity, sequence−specific DNA binding [72]
GO:0044212 Transcription regulatory region DNA binding [58]
GO:0001067 Regulatory region nucleic acid binding [59]
GO:0000976 Transcription regulatory region sequence−specific DNA binding [50]
GO:0001012 RNA polymerase II regulatory region DNA binding [45]
GO:0000977 RNA polymerase II regulatory region sequence−specific DNA binding [45]
GO:0000982 Transcription factor activity, RNA polymerase II proximal promoter sequence−specific DNA binding [33]
GO:0044424 Intracellular part [503]
GO:0005622 Intracellular [518]
GO:0043226 Organelle [482]
GO:0043227 Membrane−bounded organelle [451]
GO:0043231 Intracellular membrane−bounded organelle [390]
GO:0043229 Intracellular organelle [453]
GO:0005634 Nucleus [270]
GO:0098805 Whole membrane [76]
GO:0044853 Plasma membrane raft [10]
GO:0005901 Caveola [9]
GO:0031323 Regulation of cellular metabolic process [265]
GO:0051252 Regulation of RNA metabolic process [175]
GO:0009893 Positive regulation of metabolic process [155]
GO:0010604 Positive regulation of macromolecule metabolic process [145]
GO:0031325 Positive regulation of cellular metabolic process [145]
GO:0051173 Positive regulation of nitrogen compound metabolic process [140]
GO:0048468 Cell development [103]
GO:0006366 Transcription by RNA polymerase II [131]
GO:0006357 Regulation of transcription by RNA polymerase II [123]
GO:0045944 Positive regulation of transcription by RNA polymerase II [68]
